# Home range size and habitat quality affect breeding success but not parental investment in barn owl males

**DOI:** 10.1038/s41598-022-10324-7

**Published:** 2022-04-20

**Authors:** Robin Séchaud, Kim Schalcher, Bettina Almasi, Roman Bühler, Kamran Safi, Andrea Romano, Alexandre Roulin

**Affiliations:** 1 Department of Ecology and Evolution, University of Lausanne, Building Biophore, 1015 Lausanne, Switzerland; 2 Swiss Ornithological Institute, Seerose 1, 6204 Sempach, Switzerland; 3 Max Planck Institute of Animal Behaviour, Department of Migration, Am Obstberg 1, 78315 Radolfzell, Germany; 4 Department of Biology, University of Konstanz, Universitätsstraße 10, 78464 Konstanz, Germany; 5 Department of Environmental Science and Policy, University of Milan, Milan, Italy

**Keywords:** Conservation biology, Ecology

## Abstract

Life-history theory predicts that parents should balance their limited resources to maximize lifetime fitness, limiting their investment in current reproduction when the fitness value of current progeny is lower than that gained by producing offspring in the future. Here, we examined whether male barn owls (*Tyto alba*) breeding in low-quality habitats increased their parental effort to successfully complete offspring rearing or limited their investment by paying a fitness cost while saving energy for the future. We equipped 128 males with GPS devices between 2016 and 2020 to collect information on home range size, habitat composition, food provisioning rate to the brood and nightly distances covered. We also recorded nestlings’ growth and survival, as well as males’ body mass variation and future reproductive success. Males living in lower-quality habitats exploited bigger home ranges compared to individuals whose nests were settled in prey-rich habitats. They fed their brood less frequently, while covering longer nightly distance, resulting in a slower growth of late-hatched nestlings and ultimately in a lower fledging success. As males did not differ in body mass variation or future reproductive success our findings suggest that males hunting in home ranges with less prey-rich structures do not jeopardize future reproduction by investing disproportionately larger resources to compensate for their current low home range quality.

## Introduction

A central issue in life-history theory concerns how parents optimally balance their limited resources in order to maximize their lifetime fitness^1,2^. In species with altricial progeny, rearing offspring is among the most energetically demanding activity for the parents^3–5^. Parental investment in current reproduction is therefore expected to result in trade-offs against parental survival^6–8^ and future reproduction^9–11^. When the reproductive effort in the current breeding event results in a considerable loss of future fitness, via a decrease in survival and/or a reduction in future fecundity, the optimal parental investment is smaller than what it would be to maximize current offspring production^12,13^. In practice, whenever the cost of a reduced annual fitness could be overcompensated by a larger increase in future fitness, it can be advantageous for the parents to prudently limit their investment in current reproduction, thus resulting in brood reduction or failure^12,14^.

The successful completion of offspring rearing may depend on individual quality but also on the environmental conditions experienced by both the parents and offspring. It is well-known that parents are usually limited by food availability^15^ which is typically related to the quality and the structure of the environment where the breeding occurs and which may considerably constrain the investment in the current offspring^16,17^. In particular, when the habitat is degraded and fragmented, available resources become increasingly scattered and isolated, thus forcing individuals to maintain larger home ranges^18^ and to forage at increasing distances from their breeding site^19,20^. This has been described in different taxa, both in primary consumers^21,22^ and predators^16–18,23,24^. Furthermore, an increase in home range size has been shown to result in lower provisioning rate to the offspring^19,20^, longer distances covered^19,23,25^ and larger energetic costs to the parents^26^. The ultimate consequence may be a decrease in current reproductive success^27,28^ or survival^29^. Under limiting ecological conditions, parents have therefore to decide whether to compensate through an increase in their reproductive effort in order to successfully complete offspring rearing at the expense of their future reproduction^30–32^ or to limit their investment, decreasing their current fitness, in order to improve self-maintenance and gain future reproductive chances^4,33^. There is still a dearth of studies investigating how habitat quality can affect the amount of parental care, and how it can mediate individual trade-offs^34–36^.

To examine how parental investment in offspring rearing, reproductive success and future reproduction are affected by habitat quality, we performed a GPS tracking study on a large sample of male barn owls (*Tyto alba*), recording home range size and habitat characteristics. This species is a farmland nocturnal raptor of medium size, that preys almost exclusively on small mammals^37,38^. It breeds in farms and barns, and hunts preferentially in extensive open habitats, such as meadows and wildflower strips^39–42^. However, it can also exploit more intensive habitats, like grasslands and cereal crops, but shows a strong avoidance for forests and urbanized areas^40–42^. The vast majority of food delivered to the altricial offspring is provided by the male, especially from the second week after hatching to the moment of fledging^43^, females in contrast can even abandon their brood to start a second one elsewhere^44^.

Here, we investigated how habitat composition and home range size, as proxies of habitat quality, affected male hunting behaviour and parental investment measured as food provisioning rate, nightly distance covered, and body mass variation during the rearing period. We also measured how parental effort translated into offspring growth and pre-fledging survival, as well as affected future reproduction. In particular, we predicted that home range size should decrease with increasing proportion of high quality habitats surrounding individual nests^16,18,45^. We consequently expected that male food provisioning rate should increase with decreasing home range size, while the opposite should be the case for nightly distance covered. In practice, we expected males maintaining larger home ranges to pay higher costs of reproduction. We then tested two competing hypotheses about male investment in current reproduction. Under the “compensation hypothesis”^31^, males living in low-quality habitats would increase their effort in order to maintain nestlings’ survival and maximize current reproductive success, while paying the associated larger energetic cost including a lower probability to breed during in the following breeding season. If this hypothesis is met, we expected a reduction in body mass and body condition^6,46,47^ for males living in large home ranges (i.e. low-quality habitats) in order to provide the amount of food needed to successfully accomplish the rearing of all their nestlings. Such an increased reproductive effort should result in a comparable number of offspring reared compared to males living in small home ranges, but a smaller probability of reproduction and a lower reproductive success in the following breeding season. Conversely, under the “prudent father hypothesis”^4^, males in low-quality habitats would limit their efforts not to compromise future reproduction. If this hypothesis is met, we expected that males living in low-quality habitats should not pay costs in terms of reduced body mass and condition as well as of future reproduction, but in terms of reduced current reproductive success (i.e., brood reduction).

## Materials and methods

### Study area and species

The study was performed between 2016 and 2020 in an area located in Western Switzerland, in a typical farmland landscape. Intensive crops cover the majority of the area, interspersed with villages and forests^48^. Recently, agri-environment schemes (AES) were implemented in the landscape to maintain and promote biodiversity, including mainly extensively exploited meadows and pastures, wildflower strips and hedges. These areas host high densities and diversity of small mammals compared to surrounding intensively exploited crops, as shown by specific surveys which are periodically performed in the study area^39,49^, and barn owls use them preferentially when hunting^42^.

Nest boxes for barn owls have been installed in the study area since 1985 to counter the loss of natural breeding sites. Barn owl females produce an egg every 2–3 days and start incubating them as soon they are laid, resulting in a hatching asynchrony of several days between each nestling. Barn owls lay 6 eggs on average (from 1 to 11), from which 4 fledglings (from 0 to 9) are raised successfully^48^.

Importantly, we focused on males only because of several reasons. First, post-hatching parental investment varies between sexes, with males being the main prey providers (three quarters of the prey on average^43,50^). Second, the female, in addition to being little involved in feeding the nestlings, may desert the brood to produce another clutch elsewhere (up to 59% of the females in certain years^44^). This causes them to travel great distances in search of a free nesting site, which they typically find relatively far from their first nest (4.6 km on average, but up to 29.1 km^44^). Third, even if females do not desert their nest, they display a wide inter-individual variation in behaviour that still require further studies, with some owls spending their nights perching close to the nest while others wandering about for most of the time. Thus, at the time of our study, the home range of females might depend on many other factors than habitat quality, and we therefore did not consider them in our analyses as it was impossible to establish the relationship between their home range size and the reproductive success.

### GPS tag deployment

Breeding males were captured at their nest site when the oldest nestling was 19 to 30 days old (mean of 24.8 days), using a well-established procedure (authorizations of the Department of the consumer and veterinary affairs: VD and FR 2844 and 3213; Séchaud et al., 2021). By deploying the GPS tags at the same nestling age, we ensured that the male’s home range size was not affected by the age of the nestlings (Estimates (SE) = − 0.076 (0.177), *p* = 0.668; lmer with the year set as random term).

The males were equipped with small GPS devices fixed on their back with a Teflon harness. In 2016 and 2017, we used GiPSy-5 tags (Technosmart, Italy) programmed to collect location every 10 s. In the three following years, we used Axy-Trek tags (Technosmart, Italy), with a 10-s interval sampling rate in 2018, and a 1-s interval in 2019 and 2020. In the present study the data collected in the two last years were down-sampled to 10 s to match the previous data. Both type of tags weighed approximately 12 g including the battery and were packed in a protective plastic sheath for a final size of 30 × 20 × 10 mm, with an additional 40 mm long antenna. Since barn owls are strictly nocturnal in Switzerland, we increased the GPS battery lifespan by switching the tags to standby during the day. The owls were recaptured on average 11 days later (range: 6 to 22 days). In total, we obtained 161 GPS tracks (32 in 2016; 18 in 2017; 40 in 2018; 39 in 2019; 32 in 2020), recording on average for 8 nights (range: 4 to 14), from 128 males (106 tracked once, 12 twice, 9 thrice and 1 four times). Prior to any analysis, GPS data were filtered for aberrant positions using speed (excluding locations with a speed higher than 15 m/s) and location (excluding locations outside the study area due to GPS errors). The final data set included 2′307′236 locations (out of the 2′309′883 collected in total).

### Home range size and composition

For each individual, we estimated the 95% kernel home range using the *ctmm* R package^51^ to account for the temporal auto-correlation present in our datasets^52^. To calibrate the *ctmm* model, we placed a GPS device on a pole in open landscape and used the data collected as User Equivalent Range Error (UERE). The model best fit was chosen automatically with the *variogram.fit* function in the same package. Variogram plots were then visually inspected, and the home range size extracted.

To investigate the quality of the habitat exploited by individual barn owls, we looked at the relationship between home range size and the proportion and diversity of AES. The AES were specifically implemented in farmland to promote biodiversity, and have been shown to be preferentially used by hunting barn owls^42^. A vole monitoring in the study region in the years 2015–2021 showed that AES contain a higher abundance of voles (mean number of heaps, holes and runways along 5 m transects: 1.5 ± 3.5 (SD), range 0.6–3.5, n = 1856 transects) compared to intensive meadows (0.4 ± 1.9, range 0.1–0.7, n = 6024) or winter cereal fields (0.3 ± 1.7; range 0.1–0.6, n = 7143), which was also found by^39,49^. AES surveys were obtained from the Department for Agriculture, Viticulture and Veterinary Affairs of the Vaud canton and the Department for Institutions, Agriculture and Forestry of the Fribourg canton, and were only available for the years 2018 to 2020. Among the 26 AES types present in the study area (Table S1), we excluded the less abundant ones (representing < 1km^2^), as well as the AES types specific to a small region of the study area (which were available to only a few breeding pairs). The six remaining AES types were grouped in four main categories—extensive meadows, extensive pastures, wildflower strips and hedges–representing 93% of the AES surface implemented in the study area (Table S1). These six AES types constitute the vast majority of the AES present in the study area, and can be found in all of it, thus representing the major hunting grounds for the barn owls. For the analyses, we used the proportion of AES present in the home range, as well as its Shannon Diversity Index (hereafter called AES diversity) estimated using the *vegan* package^53^.

### Breeding and individual parameters

During the breeding season, the nest boxes were visited every month to find the ones occupied by breeding pairs. Once a clutch was found, we followed a standardized protocol of visits to the nest to record the following breeding parameters: number of eggs, nestlings and fledglings^48^. The number of eggs was recorded a week before hatching, ensuring that all eggs were laid. The number of nestlings was recorded both at the installation and recovery of the GPS (see *GPS tag deployment* above), as well as their wing length to estimate growth during this period. To account for differences in timespan between installation and recovery of the GPS among males, we calculated a daily wing growth rate by dividing the increase in wing length by the time elapsed between the two measurements for each nestling. Wing length was preferred to body mass as the latter can vary considerably with the recent consumption of a prey (e.g., the weight of a prey can reach up to 50% of the body mass of a nestling). We considered as “fledgling” all nestlings that reached 55 days of age, which corresponds to their first flights out of the nest^48^.

We measured body mass and wing length of the males at both capture sessions (GPS deployment and recovery). As adults, their wing size does not change during the breeding season, so we estimated a daily body mass variation by dividing the difference in weight by the number of days between the two capture events. Body mass variation is a commonly used proxy of parental investment in bird studies (e.g. 6,46,47). For each individual, we recorded its age based on ringing information (if it was previously ringed as nestling or as adult in the previous years) or feather moulting pattern (distinguishing yearlings from old birds^54^). Then, as not all birds could have been aged precisely, we classified them in two age groups, representing their previous breeding experience: yearlings (i.e., unexperienced) or old (i.e., experienced) individuals. Although many birds breed at one year old, some might reproduce for the first time at an older age.

To measure the long-term effects of habitat quality on males, for each individual we recorded the probability to breed and its reproductive success in the year following the one when GPS was deployed. It is unlikely that males leave the study area as almost only young individuals disperse, while breeding philopatry is common^55^. However, barn owls regularly change nesting sites between years^56^. The reproductive success of the following year, measured as the number of fledglings produced in the first clutch (see above for details), was available for a subset of 56 males.

### Movement parameters

We measured the average nightly distance covered by each GPS tagged bird as the mean of the sum of the distances between consecutive GPS locations per night. We excluded the night of GPS installation (as the bird behaviour might have been altered by the capture) and the last night if not recorded completely. Perching locations were excluded from the estimation of the nightly distance covered, as, when birds perch, the GPS locations differ slightly and could generate wrong distances by accumulating GPS error^42^. To this purpose, we used the Expectation–Maximization binary Clustering (EMbC) method implemented in the *EMbC* package to identify behaviour modes^57^. EMbC clusters movement data based on speed and turning angle between locations. Perching, due to the small GPS location errors, was characterized by low speed and a wide range of turning angles, while movement was characterized by medium to high speed and medium to high turning angles^42^. We compared EMbC classification with a visual classification of perching locations and found an average match of 94.5% (SE = 2.3; San-Jose et al., 2019).

As a single prey is brought per nest visit^43^, we estimated the average nightly prey provisioning by counting the number of visits to the nest box per night. The visits were identified using the *recurse* package^59^ by setting a radius of 150 m around the nest site and ignoring all excursions outside of the radius for less than 60 s. Using this procedure, we found an average prey provisioning of 8.8 prey items delivered to the nest per night, which corresponds to the previous feeding rate reported for barn owls^43,50^. To further validate the method, we compared the visits obtained with the GPS tracks to visits assessed with camera traps installed in front of 10 nests. We found that 98.3% (range: 95.2–100%) of the visits correctly corresponded to feeding events, and thus considered this method as highly reliable to assess prey provisioning.

### Statistical analyses

#### Home range size and habitat quality

We modelled the effects of home range composition, as well as individual and temporal parameters on the home range size using a linear mixed-effect model in *lme4* package^60^, and corresponding p-values were obtained using *sjPlot* package^61^. In all models, numeric covariates were standardized (z-transformed). The proportion and diversity of AES in the home range were included in the model as predictors of the home range size. Because the AES official mapping started in 2018, no data were available for the two first years of our study (2016 and 2017). Hence, in this first model, we only considered the owls tagged from 2018 to 2020 (n = 127), but as the surfaces of the different AES categories were similar between years, we expected to observe similar patterns in the previous years (Spearman’s correlation = 0.99; Table S1). Individual’s age category (yearling or old) and the laying date were also included as covariates, and the year of the observation was set as random factor. Considering that 22 individuals were captured and deployed with GPS in multiple years (see above) individual identity (hereafter individual ID) was also added as random factor to all the models. However, to check whether repeated measures of the same individuals would have affected the results, all the models were re-run using a single datum per individual (without individual ID as an additional random factor). These analyses always provided qualitatively similar results (details not shown for brevity), and therefore in the main text we report the output of the models including the largest sample size. For this and all linear mixed-effect models, we first checked for collinearity between predictors and then verified the model assumptions by visually inspecting residual diagnostic plots.

#### Reproductive success and nestling growth

We investigated breeding success in relation to home range size at different development stages of the clutch (n = 161), namely number of eggs (square root transformed) and fledglings, using linear mixed-effect models. The laying date, and the male’s home range size and age (yearling or old) were included as covariates. The year of observation and the individual ID were included as random factors. The “fledglings” model also included the number of eggs laid as a covariate. Then, using the same covariates and random factors as for the “fledglings” model, we modelled the fledging success by comparing the ratio between the number of eggs that succeeded and failed to fledge using generalised linear mixed-effect models with a binomial distribution.

Finally, to study the daily nestling wing growth rate (between GPS installation and recovery, n = 592 individuals), we fitted a model including laying date, the brood size, the nestling rank in the brood age hierarchy (continuous variable; rank number 1 is assigned to the oldest nestling), and the home range size and age of the father as covariates. We included the interaction between the home range size and the nestling rank as home range size might differently correlate with early- or late-hatched nestlings growth rate. Brood identity and year were included as random factors. As high ranks can be found only in large broods, we ran the same analysis considering only broods with a maximum of 5 nestlings in order to check that these results were not affected only by the presence of few very large broods.

#### Parental investment

To study potential mechanisms explaining the effect of habitat quality on fitness, we looked at the father’s average nightly prey provisioning rate, average nightly distance covered and body mass variation (n = 161 GPS tracks; 128 males). The “prey provisioning rate” was square root transformed, and the “distance covered” log transformed, to meet the model assumptions. All three models included laying date, home range size, age and the number of nestlings as covariates, while the year of observation and individual ID were set as random factors.

#### Probability to breed and future reproduction

To examine the effect of habitat quality and the reproductive effort on the males’ long-term fitness, we investigated their probability to breed and their future reproduction success (i.e., number of fledglings produced) the year following the one when the GPS was deployed. We modelled the “probability to breed” using generalised linear mixed-effect models with a binomial distribution, and the “future reproduction” using linear mixed-effect models. Both models included laying date, home range size, male age and the number of nestlings as covariates, while the year of observation and individual ID were set as random factors.

## Results

### Home range size and habitat quality

The home range size of the male barn owls tracked ranged from 1.1 to 19.8 km^2^ (mean = 6.0 km^2^; SD = 3.7). We found that smaller home ranges contained higher proportion of AES than bigger ones. In contrast, bigger home ranges included a higher Shannon diversity of AES. We did not detect any effect of male age or laying date on the size of home ranges (Table 1).

**Table 1:** Male home range size in relation to male age, laying date and home range AES composition.

Predictors	Estimates (SE)	*t*	*P*
(Intercept)	1.651 (0.113)	14.644	** < 0.001**
Male age (old)	0.007 (0.111)	0.059	0.953
Laying date	− 0.047 (0.058)	− 0.823	0.412
AES proportion	− 0.137 (0.062)	− 2.201	**0.030**
AES diversity	0.182 (0.061)	2.970	**0.004**

Results of a linear mixed-effect model with the year of observation and the individual identity set as random factors, including 127 home ranges measured between 2018 and 2020. Home range size was log-transformed. Standardized estimates (z-transformed) are provided. AES stands for agri-environment schemes, habitat types implemented in the study area to promote biodiversity. Significant values are highlighted in bold.

### Reproductive success and nestling growth

**Figure 1:**
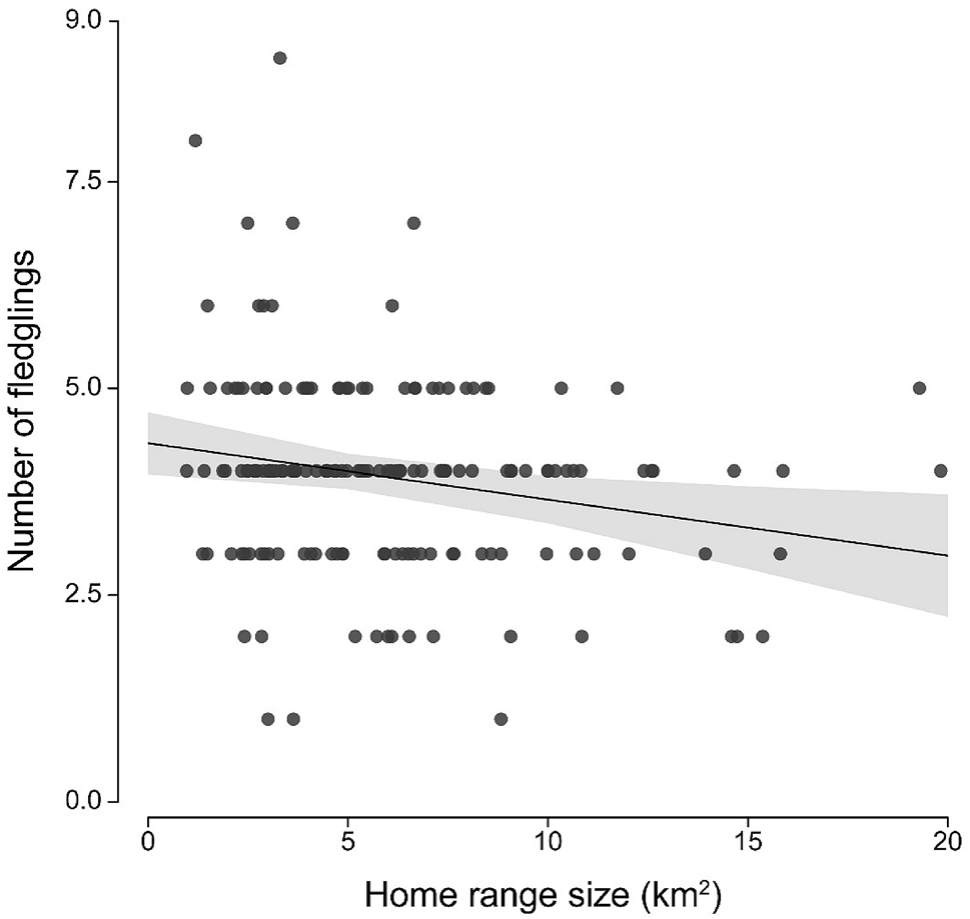
Number of fledglings produced in relation to male home range size (n = 161). The continuous line represents the predicted number of nestlings in relation to male home range size, and the grey area the 95% confidence intervals associated (from the model reported in Table 2).

Although the number of eggs laid was not related to home range size, the number of fledglings and the fledging success were higher for males with smaller home ranges (Table 2; Fig. 1). The number of eggs increased with laying date, while we could not detect any effect of laying date on the number of fledglings and fledging success. We did not find any effect of male age in any of the models (Table 2).

**Table 2:** Number of eggs, number of fledglings and fledging success in relation to male home range size, male age and laying date.

Predictors	Number of eggs			Number of fledglings			Fledging success		
	Estimates (SE)	*t*	*p*	Estimates (SE)	*t*	*P*	Estimates (SE)	*t*	*p*
(Intercept)	2.518 (0.052)	48.147	** < 0.001**	3.839 (0.145)	26.428	** < 0.001**	0.579 (0.109)	5.288	** < 0.001**
Male age (old)	− 0.023 (0.048)	− 0.488	0.627	0.155 (0.204)	0.758	0.450	0.116 (0.147)	0.790	0.430
Laying date	0.078 (0.023)	3.341	**0.001**	− 0.014 (0.103)	− 0.131	0.896	− 0.006 (0.072)	− 0.079	0.937
Home range size	− 0.026 (0.023)	− 1.172	0.243	− 0.214 (0.097)	− 2.201	**0.029**	− 0.166 (0.073)	− 2.281	**0.023**
Number of eggs				0.261 (0.101)	2.582	**0.011**	− 0.424 (0.074)	− 5.719	** < 0.001**

Results of two linear (number of eggs and number of fledglings) and one generalised linear (fledging success) mixed-effect models with the year of observation and the individual identity set as random factors, including 161 home ranges measured between 2016 and 2020. The number of eggs was square root transformed, and the fledging success model compared the number of eggs laid to the number of fledglings produced. Standardized estimates (z-transformed) are provided. Significant values are highlighted in bold.

**Figure 2:**
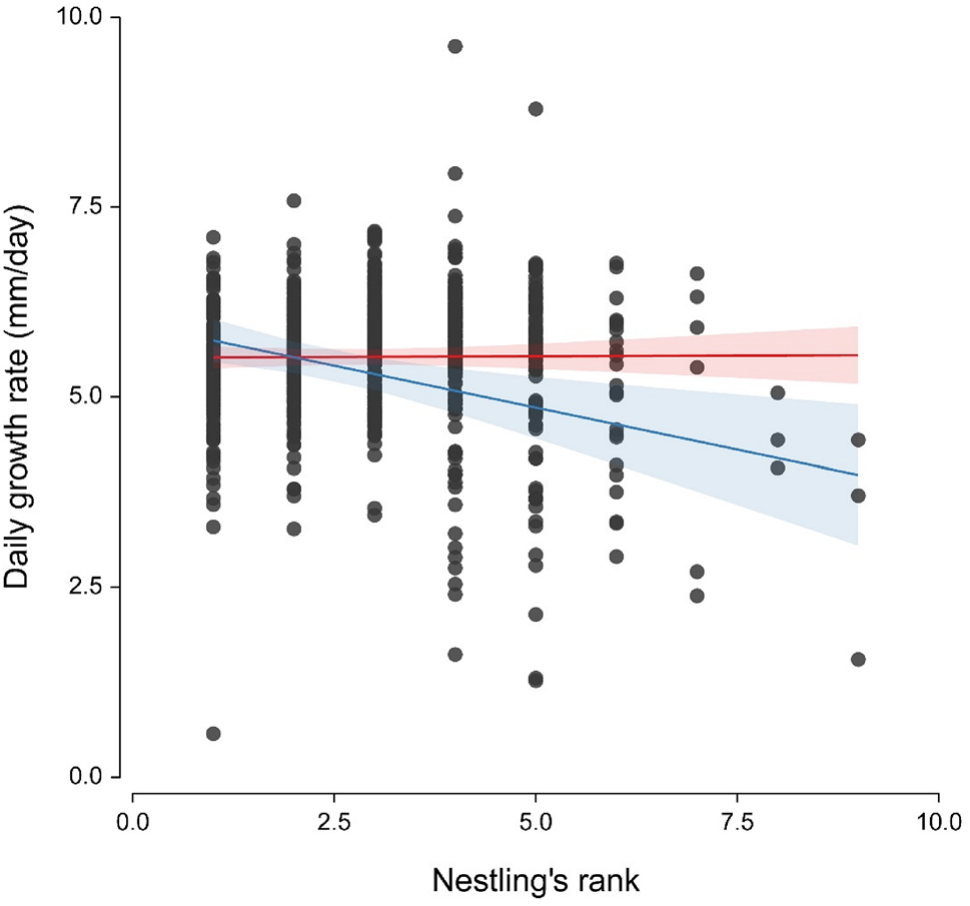
Nestling daily growth rate (n = 592 nestlings) in relation to its rank and male home range size. The continuous lines represent the predicted nestling’s growth rate in relation to its hatching rank, and the shaded areas the 95% confidence intervals associated (from the model reported in Table 3). The red line represents the smallest (1.0 km^2^) and the blue line the biggest (19.8 km^2^) home ranges, respectively. This division was arbitrarily chosen to facilitate the visualisation of the result.

When investigating nestling wing-length growth, we found a significant effect of the interaction between nestling rank and home range size (Table 3). Specifically, while growth rate of early-hatched nestlings was similar in broods reared by males with different home range size, late-hatched nestlings reared by males with large home range size suffered from a slower growth compared to those reared by males maintaining small or intermediate home ranges (Fig. 2). In addition, we did not observe any effect of neither laying date nor male age or the number of nestlings on nestling growth (Table 3). The complementary analysis considering only broods with a maximum of 5 nestlings (to account that high ranks can only be found in large broods) presented similar results (Table S2).

**Table 3:** Nestling growth rate in relation to its position in the brood age-hierarchy (rank), male home range size, male age and laying date.

Predictors	Estimates (SE)	*t*	*P*
(Intercept)	5.491 (0.058)	94.809	** < 0.001**
Male age (old)	− 0.036 (0.081)	− 0.448	0.655
Laying Date	0.019 (0.040)	0.480	0.632
Home range size	− 0.059 (0.039)	− 1.533	0.127
Number of nestlings	− 0.042 (0.041)	− 1.030	0.305
Nestling’s rank (Rank)	− 0.085 (0.034)	− 2.470	**0.014**
Home range size × Rank	− 0.102 (0.039)	− 2.619	**0.009**

Results of a linear mixed-effect model including 740 nestlings, with the year of observation and the brood identity set as random intercepts. Standardized estimates (z-transformed) are provided. Significant values are highlighted in bold.

### Parental investment

**Figure 3:**
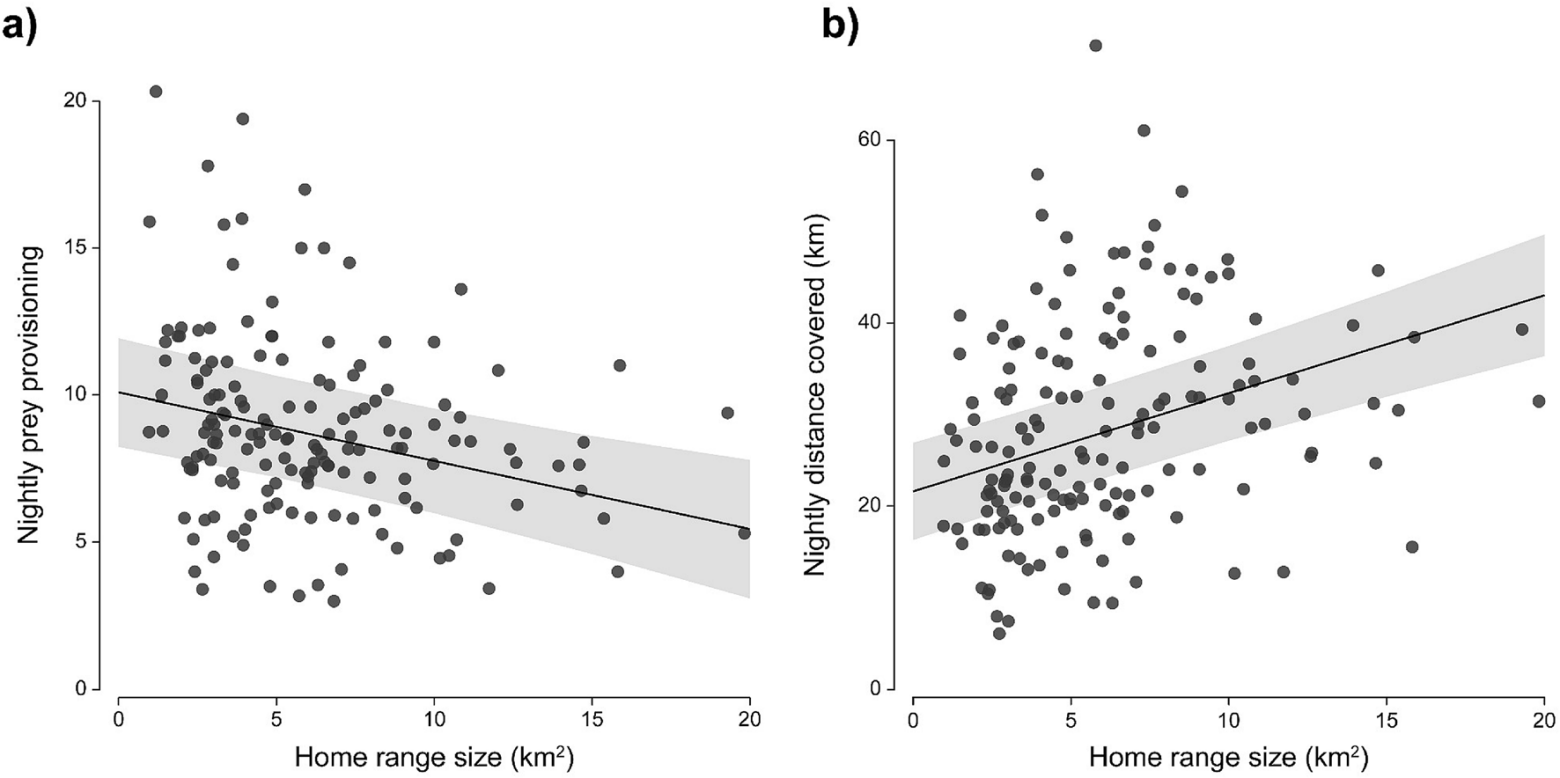
Male prey provisioning rate and nightly distance covered in relation to their home range size. The continuous lines represent a) the predicted prey provisioning rate and b) the predicted distance covered per night in relation to male home range size, and the grey area the 95% confidence intervals associated (from the models reported in Table 4).

Home range size correlated negatively with nightly prey provisioning rate and positively with nightly distance covered (Fig. 3; Table 4). Thus, males with smaller home ranges provided more prey to the nest, while covering shorter distances than males with larger ones. However, home range size did not explain male body mass variation. Brood size predicted positively nightly prey provisioning rate and distance covered, but not the male body mass variation (Table 4). Laying date did not affect prey provisioning nor male body mass variation, but the distance covered significantly decreased with laying date although the effect was small. Male age was not statistically significant in these models.

**Table 4:** Male average nightly prey provisioning rate, average nightly distance covered and daily body mass variation in relation to its home range size, age, laying date and brood size.

Predictors	Prey provisioning			Distance covered			Body mass variation		
	Estimates (SE)	*t*	*p*	Estimates (SE)	*t*	*p*	Estimates (SE)	*t*	*p*
(Intercept)	2.922 (0.160)	18.269	** < 0.001**	3.285 (0.100)	32.985	** < 0.001**	− 0.500 (0.620)	− 0.806	0.422
Male age (old)	− 0.049 (0.074)	− 0.660	0.510	− 0.076 (0.045)	− 1.692	0.093	− 0.076 (0.319)	− 0.237	0.813
Laying date	0.031 (0.036)	0.840	0.402	− 0.054 (0.022)	− 2.440	**0.016**	0.206 (0.159)	1.297	0.197
Home range size	− 0.119 (0.035)	− 3.366	**0.001**	0.159 (0.022)	7.186	** < 0.001**	0.035 (0.152)	0.232	0.817
Brood size	0.113 (0.036)	3.184	**0.002**	0.229 (0.025)	9.256	** < 0.001**	0.139 (0.115)	1.212	0.227

Results of linear mixed-effect models with the year of observation and the individual identity set as random intercepts, including 161 home ranges measured between 2016 and 2020. The prey provisioning rate was square root transformed, and the distance covered was log-transformed. Standardized estimates (z-transformed) are provided. Significant values are highlighted in bold.

### Probability to breed and future reproduction

Home range size did not predict the probability to breed in the following year nor the future reproductive success (Table 5). None of the other variables (male age, laying date, and brood size) included in the models showed a significant effect.

**Table 5:** Male probability of breeding in the year following the one when the GPS was deployed and future reproduction (number of fledglings produced the next year) in relation to its current home range size, age, laying date and brood size.

Predictors	Probability to breed			Future reproduction		
	Estimates (SE)	*t*	*p*	Estimates (SE)	*t*	*p*
(Intercept)	− 1.433 (1.191)	− 1.203	0.229	5.250 (0.423)	12.422	** < 0.001**
Age (old)	0.624 (0.576)	1.083	0.279	0.144 (0.507)	0.285	0.776
Laying date	− 0.009 (0.296)	− 0.030	0.976	0.328 (0.245)	1.336	0.181
Home range size	0.412 (0.281)	1.464	0.143	− 0.023 (0.241)	− 0.094	0.925
Brood size	− 0.026 (0.276)	− 0.094	0.925	− 0.159 (0.233)	− 0.683	0.495

All predictors (age, laying date, home range size and brood size) relate to the current reproduction, whereas response variables (probability to breed and future reproduction) to the following year. Results of a generalised linear mixed-effect models with a binomial distribution (probability to breed model) and a linear mixed-effect model (future reproduction model, measured as the number of fledglings produced the next year) with the year of observation and the individual identity set as random intercepts, including respectively 129 and 56 home ranges measured between 2016 and 2019. Standardized estimates (z-transformed) are provided. Significant values are highlighted in bold.

## Discussion

In the present study, we investigated how the quality of breeding environment, as gauged by home range size, predicted individual reproductive success and parental effort in a large number of barn owl males. We found that individuals breeding in high-quality habitats, which include a large proportion of prey-rich AES habitats, maintained smaller home ranges than individuals in low quality habitats. Not surprisingly, these habitats are associated with a large presence of the main prey of the barn owl in the study area^39,49^. This is consistent with a large body literature on birds and mammals, spanning from primary consumers^21,22^ to top predators^24^, including other raptor species^16–18,23,62^. In practice, in high-quality habitats organisms are able to find more resources in the proximity of their breeding site, without the need to cover long distances to hunt, which may result in a smaller energetic and metabolic expenditure compared to those individuals that are forced to forage further^63,64^. This is particularly important during the rearing of altricial offspring, which need a large food supply to be sustained until fledging. We also found that males having large home ranges cover a larger distance every night in order to provide food to their broods, which, however, are fed less frequently than those reared in high-quality habitats. A reduced feeding rate corresponds to fewer food items received, since barn owl parents provide a single prey item only per feeding visit. The reduction in feeding rate was the main candidate to explain why the fledging success of broods reared by males in large home ranges was smaller despite equal numbers of eggs laid. Several previous studies showed a negative relationship between home range size/habitat quality and feeding rate^18,19,23,25^, as well as reproductive success^18,23,65^ and distance covered^19,23,25^.

We also showed that the reduced fledging success is likely mediated by the death of the smallest nestlings of the broods raised by males with large home ranges. Indeed, although growth rate of the early-hatched nestlings was similar among broods, that of the late-hatched nestlings was considerably faster in broods reared by males with high-quality home ranges compared to those reared by males with low quality home ranges. Such an observation suggests that under food shortage fathers may favour the offspring of higher reproductive value^36,66^ and/or, more likely, larger and highly-competitive nestlings may monopolize the scarce resources to the detriment of their smaller siblings, as commonly observed in avian species^67–69^, including barn owls^70^. Therefore, males maintaining small high-quality home ranges were able to rear a larger number of nestlings, without apparently incurring increased energetic costs. This was not the case for males hunting in large home ranges, which, because of the large distance they had to cover, would have had to considerably increase their hunting effort in order to provide enough food for all their offspring to successfully fledge. However, we could not detect any increased costs in terms of body-mass variation during rearing in males with large home-ranges. When breeding in low-quality habitats, barn owl males seem not to increase their hunting effort to compensate for the lower prey abundance, and thus pay a cost in annual reproductive success (i.e., smaller number of fledglings), possibly saving their limiting energies for future reproduction. An alternative interpretation is that males breeding in low-quality habitats, despite covering more distance in search of food, are not able to find enough prey to rear all their nestlings successfully. This last hypothesis seems less likely as we would have expected a negative effect on the males’ body mass variation during this period. We thus interpret this finding as an evidence consistent with the “prudent father hypothesis”, with males trading-off their current versus future breeding opportunities. This argument is also compatible with a previous study of the same population, where an experimental increase in brood size resulted in smaller nestling growth and pre-fledging survival, but in a lack of any long-term effect on parental fitness^43^. In many birds of prey, when broods require an extra parental effort, for example due to a suboptimal environmental condition (this study) or an increased number of nestlings to be fed^23,43,71,72^, parents do not jeopardize their future reproduction, and do accept a brood reduction. An additional piece of evidence in line with the above reasoning is that males breeding in large home ranges did not pay any long-term cost in terms of future breeding opportunities, as they have the same likelihood of reproducing as well as a similar reproductive success than the other males in the breeding season following the one for which we measured breeding habitat quality.

The correlative nature of the present study prevented us from inferring causality and some of the obtained results could be partly affected by confounding factors. Indeed, without an experimental manipulation linking food abundance and home range size^16^, we can only hypothesize that variation in home range size, annual fitness and reproductive investment were due to prey availability in different habitats. In addition, considering that male previous breeding experience (i.e. age) did not predict any of the variables under investigation (including fledging success and the probability of reproducing in the following breeding season), we cannot rule out the possibility that intrinsic individual quality might have affected both hunting ability and annual fitness, with high-quality individuals being more efficient in capturing prey in the proximity of their nest and feed their brood more frequently, or simply being better able to occupy a good-quality breeding habitat. However, preliminary analyses failed to find associations between male phenotypic traits previously associated with quality and home range size (e.g., wing length and plumage colour; details not shown), thus making this possibility an unlikely one. However, even if our results would have been affected by individual quality, we note that the relationships between habitat quality, parental effort and reproductive success are still maintained. Finally, although female contribution to nestling feeding is much lower than that of males^43^, with the present data we could not account for maternal effort in hunting, and therefore in fledging success. On the one hand, females might have contributed more to parental care in low-quality habitats in order to compensate the lower male feeding rate^73–75^. Under such circumstance, female behaviour would have therefore masked the observable effects of habitat quality, thus making our results very conservative. On the other hand, females might have contributed more to feed nestlings in good-quality habitats, because of a lower cost of providing food to their broods. In such a case, a larger female investment would have exacerbated the effects. However, an eventual increase of maternal effort would have been a consequence of habitat quality in the surrounding of the nest.

A final consideration is that, irrespectively of the mechanisms determining reproductive success, from a conservation point of view, our results show that AES, adopted to limit the strong decrease in farmland biodiversity, seems to be beneficial for the barn owl. Interestingly, while high quality home ranges (i.e., small home ranges) contained a higher proportion of AES than low quality ones, the AES diversity was larger in the latter. Although this is a matter of speculation, this last result can be explained by the scarcity of the AES in the landscape, implying that larger home ranges are more likely to contain different AES types. Generally, these results are novel findings and important for conservation actions beyond the species studied here. Indeed, despite documented positive effects on plant, insect and small mammal density and species richness^76,77^, the effects of AES for larger vertebrates remained uncertain. The present results therefore enforce the conviction that proper conservation policies involving stakeholders (i.e., farmers) can have a positive effect on the entire natural communities.

In conclusion, this study, performed on a large sample of individuals, showed that habitat quality, gauged by the proportion of agri-environment schemes (AES), affects annual reproductive success and individual trade-offs in the barn owl. Males breeding in low-quality habitats did not increase their parental investment to compensate for the lower prey abundance near the nest, and, by favouring brood reduction, traded-off their current fitness against future breeding opportunities.

## Supplementary Information

Supplementary Information.

## Data Availability

The GPS datasets analyzed in the current study are available in Movebank (www.movebank.org), under the project named “Barn owl (Tyto alba)” (ID 231,741,797).
