# Supplementary material for: Home range size and habitat quality affect breeding success but not parental investment in barn owl males
Source: Sci Rep. 2022 Apr 20;12:6516. doi: 10.1038/s41598-022-10324-7 (PMC9021228; doi:10.1038/s41598-022-10324-7)
Supplement: Supplementary file 1 — Supplementary Information. [file 41598_2022_10324_MOESM1_ESM.docx]

Table S1: List of the 26 AES types present in the study area, with their respective surface in Km^2^ during the three years of survey. The most abundant ones (>1km^2^) were grouped in four categories - extensive meadows, extensive pastures, wildflower strips and hedges. The AES types 921 (high-stem orchard) and 717 (extensive vineyards) were excluded from the analyses as they are not present in the entire study area.

| **AES identifier** | **AES  description** | **AES category** | **Surface in 2018** | **Surface in 2019** | **Surface in 2020** |
| --- | --- | --- | --- | --- | --- |
| 611 | extensive meadow | extensive meadows | 71,756 | 71,481 | 72,367 |
| 617 | extensive pasture | extensive pastures | 23,178 | 23,456 | 23,550 |
| 612 | low-intensity meadow | extensive meadows | 8,252 | 8,365 | 8,348 |
| 852 | hedgerow and grove | hedges | 5,138 | 5,209 | 5,288 |
| 556 | floral fallow | wildflower strips | 5,041 | 5,106 | 5,230 |
| 921 | high-stem orchard | - | 3,949 | 4,008 | 3,995 |
| 717 | extensive vineyard | - | 2,404 | 2,415 | 2,631 |
| 557 | rotational fallow | wildflower strips | 0,999 | 1,009 | 1,029 |
| 924 | isolated indigenous tree | - | 0,620 | 0,634 | 0,630 |
| 851 | littering surface | - | 0,504 | 0,479 | 0,461 |
| 559 | herbaceous vegetation strip | - | 0,382 | 0,378 | 0,348 |
| 634 | riparian meadow | - | 0,122 | 0,126 | 0,149 |
| 904 | wet ditch, swamp, pond | - | 0,083 | 0,095 | 0,143 |
| 694 | region-specific grassland | - | 0,075 | 0,056 | 0,134 |
| 618 | wooded pasture | - | 0,014 | 0,029 | 0,119 |
| 922 | walnut tree | - | 0,131 | 0,115 | 0,116 |
| 572 | flower strip | - | 0,112 | 0,127 | 0,112 |
| 55502 | extensive cereal strip | - | 0,075 | 0,095 | 0,083 |
| 594 | region-specific open land | - | 0,012 | 0,057 | 0,040 |
| 905 | ruderal surface, spoil heap | - | 0,008 | 0,006 | 0,013 |
| 55501 | extensive oilseed strip | - | 0,002 | 0,000 | 0,004 |
| 693 | region-specific pasture | - | 0,002 | 0,002 | 0,002 |
| 908 | region-specific AES type | - | 0,003 | 0,003 | 0,002 |
| 923 | chestnut tree | - | 0,000 | 0,001 | 0,002 |
| 906 | stone wall | - | 0,001 | 0,001 | 0,001 |
| 55503 | extensive protein crop strip | - | 0,018 | 0,011 | 0,000 |
| Total |  |  | 122,881 | 123,263 | 124,796 |

Table S2: Nestling growth rate in relation to its position in the brood age-hierarchy (rank) and male home range size. Only broods containing 5 or less nestlings were considered (see Table 3 for the full analysis). Results of a linear mixed-effect model including 592 nestlings, with the year of observation and the brood identity set as random factors. Standardized estimates are provided.

| *Predictors* | *Estimates (SE)* | *t* | *p* |
| --- | --- | --- | --- |
| (Intercept) | 5.531 (0.069) | 80.354 | <0.001 |
| Age (old) | -0.082 (0.096) | -0.848 | 0.399 |
| Laying date | 0.046 (0.047) | 0.961 | 0.338 |
| Home range size | -0.038 (0.044) | -0.871 | 0.385 |
| Number of nestlings | -0.049 (0.043) | -1.124 | 0.263 |
| Rank | -0.037 (0.035) | -1.042 | 0.298 |
| Home range size * Rank | -0.118 (0.038) | -3.105 | **0.002** |
